# Older Adult Experience of Online Diagnosis: Results From a Scenario-Based Think-Aloud Protocol

**DOI:** 10.2196/jmir.2924

**Published:** 2014-01-16

**Authors:** Tana M Luger, Thomas K Houston, Jerry Suls

**Affiliations:** ^1^eHealth Quality Enhancement Research InitiativeCenter for Healthcare Organization and Implementation ResearchEdith Nourse Rogers Memorial Veteran's HospitalBedford, MAUnited States; ^2^Health Informatics and Implementation ScienceQuantitative Health SciencesUniversity of Massachusetts Medical SchoolWorcester, MAUnited States; ^3^Behavioral Research ProgramNational Cancer InstituteBethesda, MDUnited States

**Keywords:** information seeking behavior, Internet, age factors

## Abstract

**Background:**

Searching for online information to interpret symptoms is an increasingly prevalent activity among patients, even among older adults. As older adults typically have complex health care needs, their risk of misinterpreting symptoms via online self-diagnosis may be greater. However, limited research has been conducted with older adults in the areas of symptom interpretation and human-computer interaction.

**Objective:**

The intent of the study was to describe the processes that a sample of older adults may use to diagnose symptoms online as well as the processes that predict accurate diagnosis.

**Methods:**

We conducted a series of “think-aloud” protocols with 79 adults aged 50 years or older. Participants received one of two vignettes that depicted symptoms of illness. Participants talked out loud about their thoughts and actions while attempting to diagnose the symptoms with and without the help of common Internet tools (Google and WebMD’s Symptom Checker). Think-aloud content was categorized using an adapted Q-sort and general inductive approach. We then compared the think-aloud content of participants who were accurate in their diagnosis with those who were not.

**Results:**

Nineteen descriptive codes were identified from the think-aloud content. The codes touched upon Web navigation, attempts to organize and evaluate online health information, and strategies to diagnose symptoms. Participants most frequently relied on a strategy where they reviewed and then rejected the online diagnoses if they contained additional symptoms than those that were depicted in the vignette. Finally, participants who were inaccurate in their diagnosis reported being confused by the diagnosis task, lacking confidence in their diagnosis, and using their past experiences with illness to guide diagnosis more frequently than those participants who accurately diagnosed the symptoms.

**Conclusions:**

Older adult participants tended to rely on matching strategies to interpret symptoms, but many still utilized existing medical knowledge and previous illness experiences as a guide for diagnosis. Many participants also had difficulty navigating the Internet tools, which suggests an increased need for navigation aids in Web design. Furthermore, participants who were inaccurate in their diagnosis had more difficulty with the Internet tools and confusion with the task than those who were accurate. Future work in this area may want to utilize additional study design such as eye-tracking to further understand the coordination between Web navigation, online symptom information processing, and diagnostic strategies.

## Introduction

### Older Adult Health Information-Seeking and Online Self-Diagnosis

Older Internet users tend to be of higher socioeconomic status, higher education levels, and be young-old (eg, 65-70 years) rather than old-old (85+ years) [1,2]. Among older adults with Internet access (estimated to be 85% of the US population aged 50-64 years and 58% of the population aged 65 years and older), more than three-fourths seek online health information [3]. Many older adult users view the Internet as an “invaluable resource” of information that can replace the library [4,5], especially for health-related topics. In fact, some older adults claim to use the Internet to prepare for physician’s visits or better understand the information offered by their health providers [4].

Searching for information to interpret one’s own physical symptoms, or “online self-diagnosis”, is increasingly prevalent, with 35% of US adults having attempted to diagnose their own symptoms online [6]. In addition, 29% of online older adults aged 50-64 years and 13% of online older adults aged 65 years or older used the Internet to diagnose personal symptoms. Researchers and physicians have been apprehensive about patient online self-diagnosis [7-10] as online health information has been found to be of varying quality [11] and patients typically have limited health literacy or understanding [12,13], both of which could lead patients to inaccurate symptom interpretations. In addition, as older adults typically have complex health care needs, including co-morbid conditions, their risk of misinterpreting physical symptoms via online self-diagnosis may be greater. Given the safety issues that it raises and the limited research that has investigated this phenomenon, it is important to better understand the ways that older adults diagnose symptoms online.

### Models of Symptom Interpretation

Symptom interpretation and diagnostic decision-making research has naturally focused on health care professionals; however, most patients do not have the same breadth of expert knowledge to apply when interpreting their own symptoms. According to Leventhal’s common-sense model of self-regulation, a layperson interprets physical symptoms by accessing memories of past experiences with illness and general knowledge about a health concept [14-17]. Patients use this information to create a “representation” of their symptoms, which can then guide action. Similarly, work by Pennebaker [18] demonstrates that patients interpret their symptoms selectively. In other words, patients focus on physical sensations and external information, which confirm their beliefs about health and illness. This kind of reasoning stands in stark contrast to expert physicians who are thought to use “pattern recognition” or “if-then” rules to make inferences about clinical cases [19,20] and medical students who are thought to use hypothetico-deductive reasoning [21], forming an initial hypothesis based on symptoms and then collecting additional data in order to confirm or disprove their hypothesis.

Although this body of work can be used to begin to understand how patients might interpret or diagnose physical symptoms, it does not account for the additional cognitive and perceptual processes that are required to conduct and interpret an Internet search, especially for older adults [22,23]. Further, older adults may have unique needs and strategies for symptom diagnosis as a result of their unique health care needs. For these reasons, we sought to uncover the cognitive processes that older adults might use to diagnose symptoms online as well as the processes that predict accurate diagnosis.

Participants in this study engaged in a “think-aloud” procedure—talking about their thoughts and actions —while attempting to diagnose the symptoms depicted in a vignette. The think-aloud was adopted to obtain a detailed description of older adult experience with online symptom diagnosis. We utilized an adapted Q-sort [24] and general inductive approach [25] to categorize participants’ think-aloud content as well as relate the coded content to the previously outlined models of symptom interpretation. Thus, we also sought to examine whether participants relied more on past experiences with illness and prior medical knowledge (consistent with the common-sense model of self-regulation) or hypothetico-deductive reasoning to interpret physical symptoms online.

## Methods

### Study Design, Setting, and Sample

This is a human-computer interaction study that included a series of think-aloud protocols [26] conducted with 79 older adults aged 50 years or older. Participants were recruited from a registry of older adults from the counties surrounding the University of Iowa (predominantly Johnson County, Iowa). Participants were included in the study if they were (1) at least 50 years or older, (2) a community resident (ie, not living in a nursing home), (3) able to travel to the research laboratory for in-person data collection, (4) owned a computer at home, (5) did not have a previous diagnosis of dementia or cognitive impairment, and (6) did not show cognitive impairment or confusion on the Short Portable Mental Status Questionnaire (SPMSQ) [27] (ie, score ≤7), which was administered as a brief screen over the phone. Participants were told that the study was an investigation of how people search for health information. Those who participated received a US$10 community gift card in appreciation and parking vouchers for their time at the research laboratory. The study was approved by the University of Iowa Human Subjects Institutional Review Board.

### Procedure

All older adult participants received one of two illness vignettes (see Materials) and were asked to diagnose the symptoms in the vignette using one of two common Internet tools (Google search engine and WebMD’s Symptom Checker). Participants were randomly assigned to a vignette and an Internet tool in order to attempt to mitigate participant differences in computer skills and previous illness experience across the manipulated study variables.

Participants were given explicit instructions (adapted from Ericsson and Simon’s protocols) about the think-aloud before beginning the task. Participants were told to approach the think-aloud “basically like you’re talking to yourself, but loud enough for other people to hear” and that the goal was to “think-aloud as continuously as possible”. Participants were also told that the exercise would end when “you’ve come upon a diagnosis that you are satisfied with”.

The think-aloud procedure was first demonstrated by the experimenter (TML), and then the participant was given the opportunity to practice thinking out loud. When the participant felt comfortable with the procedure, he or she was given one of two illness vignettes to read and diagnose on his or her own, without any electronic aid. If the participant remained silent for five seconds, he or she was reminded to “please keep talking”. Participants were asked to choose one specific diagnosis (a specific illness or condition) in order to complete the task. No other prompting or questioning came from the experimenter regarding the diagnosis. The participant was audio-recorded during the think-aloud to allow for later transcription and analysis.

Participants then diagnosed the same symptoms, while thinking aloud, with the aid of one of two Internet tools. If the participant appeared confused or frustrated with the Internet tool for more than five seconds, the experimenter provided computer support in the form of describing the interface in more detail or describing what Web actions were available to the participant. Participants were limited to 30 minutes of search time. Most completed the task between 15 and 20 minutes. Finally, participants completed quantitative questionnaires, including demographics and computer skills (see Materials). The session typically took between one hour and two hours, depending on the participant’s interest in the tasks and speed answering the questionnaires.

### Materials

#### Illness Vignettes

Two vignettes were developed for the current study (see Multimedia Appendix 1). The vignettes depicted the symptoms of an acute health condition: mononucleosis or scarlet fever. These conditions were selected as they are rare in older adults, but still relatively common in the general population. This was to ensure that few participants would have recent experiences with the illness that could influence their diagnostic process. Participants were instructed to read the vignette as if they were experiencing the symptoms themselves. Vignettes were drafted from symptom information found at Mayo Clinic’s website [28] as well as the National Institute of Allergy and Infectious Diseases website [29]. Information was combined from multiple sites so that a Google search would not point directly to the site from which the information was drawn. Ten graduate students piloted both vignettes using both Google and WebMD’s Symptom Checker. Seven out of the ten students obtained the correct diagnosis for both vignettes.

#### Internet Tools

**Figure 1:**
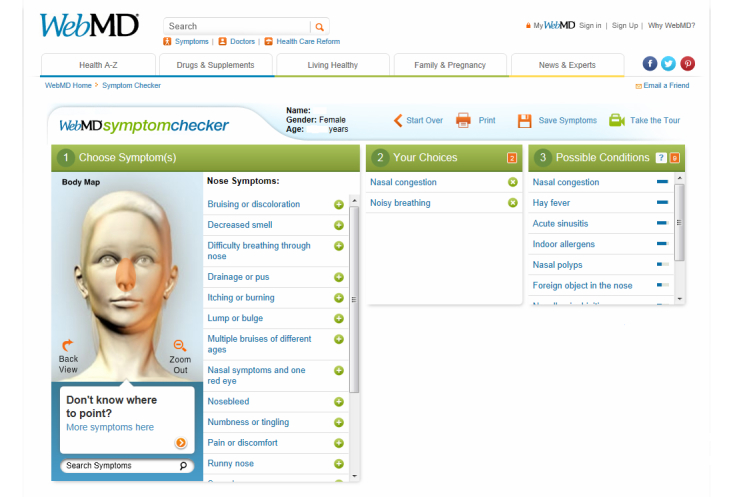
WebMD symptom checker.

The common Internet tools of Google’s search engine [30] and WebMD’s Symptom Checker [31] were employed. Google is the number one visited website in the world [32] and provides users with webpages related to search queries by ranking the relative usefulness of Internet sites [33]. WebMD’s Symptom Checker is a consumer decision aid for the purpose of self-diagnosis (see Figure 1). The application features an avatar (or pictorial representation) of the human body. In order to diagnose, the user clicks on the area of the body where his or her symptoms are located and inputs descriptors of the symptoms such as “pain”, “tenderness”, or “warm to touch”. The application then asks tailored questions based on the location of symptoms, the descriptors, and the user’s response to each previous question. After gleaning enough information, the application will present a list of potential diagnoses. The user can click on a diagnosis to get more information about its symptoms and severity as well as recommendations for care.

#### Demographics and Health

Age, gender, ethnicity, education, and income were collected via a self-report survey. Recent health history was measured using a retrospective symptom checklist [34]. Participants indicated the frequency (0 = not at all; 6 = extremely/much) with which they had experienced each of 15 symptoms (eg, headache, dizziness) in the past 21 days. To measure chronic health history, participants completed a checklist of common chronic conditions [35] (eg, diabetes, pneumonia). Participants indicated whether or not they had ever experienced each condition.

#### Computer Skills and Familiarity

Participants reported the number of hours per week that they used a home computer as well as the number of years that they had owned a home computer in order to gauge general computer familiarity. Participants were also verbally asked whether or not they had previous experience with the Internet tool to which they were assigned (Google or WebMD’s Symptom Checker). Participant responses were documented in the think-aloud audio-recording and subsequent written transcript as a yes/no response.

#### Accuracy

Participants were required to choose one specific diagnosis (ie, a specific illness or condition) in order to complete the task. Participant diagnoses were documented in the think-aloud audio-recording and subsequent written transcript. Participants were deemed to have made an accurate diagnosis if they chose the same illness as the one depicted in the illness vignette to which they were assigned (ie, mononucleosis or scarlet fever).

### Analysis: Think-Aloud Content Coding

In order to assess the design of the current study and establish an initial code list, pilot think-aloud data was collected from 15 participants aged 18 years or older at the University of Iowa Hospitals and Clinics. Audio recordings of pilot participant think-alouds (both without and using Internet tools) were transcribed into verbatim text. Each independent clause (or segment) was off-set on its own line for ease of analysis. Utilizing an adapted Q-sort method [2], the experimenter and a group of research assistants (n=9) independently read the transcript segments to identify and label categories of content/meaning. The team then met to discuss the content categories, to combine similar categories, and further refine the code list. This final version of the list was used to analyze transcripts for the current study.

In the current study, a team of 13 additional research assistants (who had not participated in the pilot work) coded segmented transcripts using the previously compiled code list. The team was instructed to label the segmented lines of the transcript with the codes that they believed were depicted. The team was advised that not every line needed to be coded and that some lines may depict more than one code. Each segmented transcript was coded by two research assistants independently in order to assess inter-rater reliability. The experimenter served as the arbitrator if there was a discrepancy in coding. Finally, the codes were examined by the authors utilizing a general inductive approach [3] to identify higher-level descriptive themes.

## Results

### Participant Characteristics

Participant characteristics can be found in Table 1. Participants were predominantly Caucasian (98%, 77/79) with an overall mean age of 63.97 years (SD 7.68). Most participants were highly educated with all having achieved some college, while 52% (41/79) had earned a post-graduate degree. In addition, most participants earned between US$50,000 and $75,000 per year (35%, 28/79). Participants were healthy, experiencing a mean of 3.11 (SD 0.55) physical symptoms in the past three weeks (out of 14 total symptoms) and a mean of 2.58 (SD 1.59) health conditions in their lifetime (out of 17 total conditions).

In terms of computer experience, the average participant had owned a home computer for almost 20 years (mean 18.17 years, SD 8.14). In addition, most participants used their home computer, on average, almost 20 hours per week (mean 18.77 years, SD 13.33). Finally, most participants had previously had experience with the Internet tool to which they were assigned (63%, 45/72).

**Table 1:** Participant characteristics (n=79).

Characteristics		n (%) / mean (SD)
Age, years		63.97 (7.23)
**Gender**		
	Male	31 (39.24%)
	Female	48 (60.76%)
**Income (USD)**		
	Less than $15,000 per year	3 (3.80%)
	$15,000-25,000 per year	6 (7.59%)
	$25,000-50,000 per year	21 (26.58%)
	$50,000-75,000 per year	28 (35.44%)
	$75,000 or more per year	21 (26.58%)
**Education**		
	Some high school	---
	High school graduate	---
	Some college	10 (12.66%)
	Associate’s degree	7 (8.86%)
	Bachelor’s degree	21 (26.58%)
	Post-graduate degree	41 (51.90%)
Number of recent physical symptoms		3.12 (2.37)
Number of lifetime health conditions		2.58 (1.59)
Years of computer ownership		18.17 (8.14)
Hours of home computer use per week		18.77 (13.33)
**Familiar with Internet Tool (n=72)** ^a^		
	Yes	45 (62.50%)
	No	27 (37.50%)

^a^Seven participants failed to respond to this interview question and were not included in analyses regarding familiarity.

### Accuracy of Diagnosis

The characteristics of accurate and inaccurate participants can be found in Table 2. Less than half of participants came to an accurate diagnosis for the illness vignette symptoms during their search (41%, 32/79). Participants who accurately diagnosed the symptoms were similar in gender, yearly income, education, years of home computer ownership, and familiarity with the Internet tool to those participants who were inaccurate in their diagnosis (see Table 2). Participants who accurately diagnosed the symptoms appeared to be slightly younger (mean 61.72 years, SD 6.17) than those who were inaccurate (mean 65.51 years, SD 7.54). In addition, those who were accurate reported an average of 3.54 (SD 2.53) recent physical symptoms and 3 (SD 1.34) lifetime health conditions as compared to the 2.83 (SD 2.23) recent symptoms and 2.28 (SD 1.69) lifetime health conditions that those who were inaccurate reported. Finally, participants who were accurate in their diagnosis used their home computers for 22.94 (SD 16.68) hours per week as compared to the 15.93 (SD 9.66) hours per week of those who were inaccurate.

**Table 2:** Participant and study characteristics by accuracy of diagnosis.

Characteristics		Accurate diagnosis (n=32) n (%) / mean (SD)	Inaccurate diagnosis (n=47) n (%) / mean (SD)
**Search Method**			
	Google	16 (50.00%)	25 (53.25%)
	WebMD Symptom Checker	16 (50.00%)	22 (46.75%)
**Illness Vignette**			
	Mononucleosis	19 (59.38%)	18 (38.30%)
	Scarlet Fever	13 (40.63%)	29 (61.70%)
Age		61.72 (6.17)	65.51 (7.54)
**Gender**			
	Male	10 (31.25%)	21 (44.68%)
	Female	22 (68.75%)	26 (55.32%)
**Income**			
	Less than $15,000 per year	3 (9.38%)	--
	$15-25,000 per year	3 (9.38%)	3 (6.38%)
	$25-50,000 per year	5 (15.63%)	16 (34.04%)
	$50-75,000 per year	14 (43.75%)	14 (29.80%)
	$75,000 or more per year	7 (21.90%)	14 (29.79%)
**Education**			
	Some high school	---	---
	High school graduate	---	---
	Some college	3 (9.38%)	7 (14.89%)
	Associate’s degree	5 (15.63%)	2 (4.26%)
	Bachelor’s degree	10 (31.25%)	11 (23.40%)
	Post-graduate degree	14 (43.75%)	27 (57.45%)
Number of recent physical symptoms		3.54 (2.53)	2.83 (2.23)
Number of lifetime health conditions		3.01 (1.34)	2.28 (1.69)
Years of computer ownership		19.66 (9.22)	17.16 (7.24)
Hours of home computer use per week		22.94 (16.68)	15.93 (9.66)
**Familiar with Internet Tool (n=72)** ^a^			
	Yes	16 (50.00%)	29 (61.70%)
	No	12 (37.50%)	15 (31.90%)

^a^Seven participants failed to respond to this interview question and were not included in analyses regarding familiarity.

### Descriptive Findings From Think-Aloud Content

#### Overview

Descriptive findings from the 19 codes are presented below and in Tables 3 and 4. From the think-aloud content, we identified three major areas related to online symptom diagnosis: (1) Internet tool navigation, (2) symptom information processing, and (3) diagnostic strategies.

**Table 3:** Think-aloud content codes and participant endorsement (n=79).

Code		Description	Percentage of participants expressing code, n (%)
**Navigation**			
	Web orientation	Comments about the layout or features of the website	70 (88.61%)
	Web navigation	Direct actions taken on the computer	78 (98.73%)
	Internet problem	Trouble or issue with the computer application	64 (81.01%)
**Symptom information processing**			
	Reading	Reading directly from the vignette or Web screen	78 (98.73%)
	Paraphrasing	Stating information found in the vignette or Web screen	79 (100.00%)
	Judgment of relevancy	Deciding whether to use information or not	72 (91.14%)
	Credibility	Discussing the source of information or trust in information	22 (27.85%)
	Confusion	Questions or statements that reflect confusion about content	44 (55.70%)
	Discussing unknowns	Talking about information that is unknown or uncertain	68 (86.08%)
	Lack of confidence	Uncertainty in a diagnosis or not knowing enough to make specific diagnosis	30 (37.97%)
**Diagnostic strategy**			
	Action plan	Stating an action that could be taken to achieve the goal of diagnosing	74 (93.67%)
	Hypothesis	Making a guess about what the diagnosis could be	78 (98.73%)
	Symptom	Selecting a specific symptom from the vignette on which to focus and search for	76 (96.20%)
	Confirmation	Matching the symptoms in the story with information about a particular diagnosis	58 (73.42%)
	Negation	A difference between the symptoms in the story and a particular diagnosis (mismatch)	72 (91.14%)
	Previous experience	Relating the symptoms or diagnosis to personal experiences	35 (44.30%)
	Previous knowledge	Relating the symptoms or diagnosis to medical information previously known	55 (69.62%)
	Cause	A potential cause of the illness (eg, a virus or germ)	41 (51.90%)
	Suggested action	Discussing potential actions for the symptoms	40 (50.63%)

**Table 4:** Think-aloud content codes by accuracy of diagnosis.

Code		Accurate diagnosis Participants expressing code (n=32)	Inaccurate diagnosis Participants expressing code (n=47)
**Navigation**			
	Web orientation	29 (90.63%)	45 (95.74%)
	Web navigation	32 (100.00%)	46 (97.87%)
	Internet problem	24 (75.00%)	40 (85.11%)
**Symptom information processing**			
	Reading	32 (100.00%)	46 (97.87%)
	Paraphrasing	32 (100.00%)	47 (100.00%)
	Cause	16 (50.00%)	25 (53.19%)
	Judgment of relevancy	27 (84.38%)	45 (95.74%)
	Credibility	9 (28.13%)	13 (27.66%)
	Confusion	16 (50.00%)	28 (59.57%)
	Discussing unknowns	28 (87.50%)	40 (85.11%)
	Suggested action	12 (37.50%)	28 (59.57%)
	Lack of confidence	9 (28.13%)	21 (44.68%)
**Diagnostic strategy**			
	Action plan	29 (90.63%)	45 (95.74%)
	Hypothesis	32 (100.00%)	46 (97.87%)
	Symptom	30 (93.75%)	46 (97.87%)
	Confirmation	24 (75.00%)	34 (72.34%)
	Negation	29 (90.63%)	43 (91.49%)
	Previous experience	13 (40.63%)	22 (46.81%)
	Previous knowledge	23 (71.88%)	32 (68.09%)

#### Internet Tool Navigation

Analysis of the think-aloud content showed that participants frequently commented on issues surrounding the use and navigation of the Internet tools. For example, participants seemed highly focused on the actions that they were taking on the computer (eg, “Type that in and hit Enter”); “web navigation” was the second most frequently identified code (14.34%, 1472/10,262) with almost all participants commenting on navigation (99%, 78/79). In addition, many participants also made comments about the layout or features of the website that they were visiting (eg, “Well, here’s a tool from the Mayo Clinic”) to orient themselves to visited websites. Finally, many participants mentioned difficulty with the computer programs, either not knowing how to navigate them or not knowing how to troubleshoot after an error message (81% of participants, 64/79; eg, “Oh, where, where did Question B go? I don’t know where Question B is. What happened there? Umm, am I at the top of Question B?”).

#### Symptom Information Processing

Participants made comments that indicated attempts to organize or evaluate the symptom and illness information encountered online. For example, participants analyzed the usefulness of the online information (eg, “Well, darn, that’s not gonna help”), followed by stating what information they were lacking (eg, “Um, but I don’t know how old this particular person is”). However, only a quarter of participants commented on the source or credibility of the online information (eg, “And the page I’m looking at, MedicineNet.com, that looks very reliable”). Diagnosing the vignette also appeared to be a difficult task for participants as about half of participants demonstrated confusion about how to attempt to diagnose the vignette symptoms (eg, “I’m kind of at a loss where to go now”). In addition, some participants also seemed hesitant to make a diagnosis and demonstrated that they were not confident in the diagnosis that they had settled upon (eg, “I can’t diagnose this by myself”).

#### Diagnostic Strategy

Participants utilized a number of strategies to attempt to diagnose the symptoms. For example, many participants planned the steps that they would take to diagnose before implementing those actions (eg, “So I guess what I will do is, uh, try to think of something that Google will be interested in trying to answer”). Many participants also focused on only one symptom at a time, inputting each symptom into the Internet tool separately, rather than attempting to diagnose the entire collection of symptoms at once (eg, “High fever…I’m going to put this in quotes”). Some participants considered the cause of the vignette symptoms (eg, “Could be bacterial, could be viral”) or suggested some sort of action that should be taken in response to the symptoms such as going to the doctor or asking for antibiotics. Participants most frequently relied on a strategy where they reviewed and then rejected potential diagnoses that contained additional symptoms than those that were depicted in the vignette (eg, “No, this person is not short of breath”). This strategy was used more than confirming potential diagnoses by comparing whether the illness/condition information contained symptoms that, in fact, matched the vignette symptoms (eg, “It fits some of it. High fever and lymph nodes”). About two-thirds of participants utilized lay/existing medical knowledge to diagnose the symptoms, even while using the computer simultaneously (eg, “Ummm, colon polyps, that’s not symptomatic”). In addition, approximately half of participants described memories of previous experiences with the symptoms and illness to aid in diagnosis (44%, 35/79; eg, “Been there, done that, um, so I had it as a kid”). However, these strategies were identified less frequently than the matching strategies discussed above.

#### Accuracy and Think-Aloud Content

We then compared the think-aloud content between participants who accurately diagnosed the symptoms and those who did not (see Table 4). Participants who inaccurately diagnosed the symptoms seemed to express more difficulty with the diagnosis task. For example, 60% (28/47) of inaccurate participants mentioned confusion about the task as compared to only half of accurate participants (50%, 16/32). Similarly, 45% (21/47) of inaccurate participants lacked confidence in their diagnosis as compared to 28% (9/32) of accurate participants. In addition, participants who were inaccurate appeared to have more difficulty navigating the computer: 85% (40/47) of inaccurate participants mentioned having an Internet problem or difficulty with the Internet tool as compared to 75% (24/32) of accurate participants.

In terms of diagnostic strategy, there was little difference between the proportion of inaccurate and accurate participants who utilized a confirmation strategy (inaccurate: 72%, 34/47 vs accurate: 75%, 24/32) or a negation strategy (inaccurate: 92%, 43/47 vs accurate: 91%, 29/32). However, inaccurate participants seemed to rely more on their previous experiences with illness (47%, 22/47) than accurate participants (41%, 13/32). In addition, inaccurate participants seemed less likely to utilize previous medical knowledge (68%, 32/47) than accurate participants (72%, 23/32).

## Discussion

### Processes of Online Self-Diagnosis

In this think-aloud protocol, the process of older adult online self-diagnosis was explored. Older adult participants frequently commented on navigating the websites visited. Participants also organized information by considering what else they would need to know or whether information encountered was useful. Most participants tended to diagnose physical symptoms through a matching process, utilizing information encountered online.

Our results most aligned with hypothetico-deductive reasoning strategies where participants utilized the additional health information available online to confirm or reject various illnesses/conditions. However, some participants also relied on existing medical knowledge to diagnose the symptoms, noting potential causes of symptoms and treatment-seeking actions, as well as recalled previous personal experiences with the symptoms, which informed their diagnosis. These findings are similar to the common-sense model.

Interestingly, more participants who were inaccurate in their online symptom diagnosis mentioned previous experiences with illness than those who were accurate. According to the common-sense model, symptom interpretation is typically undertaken with the help of heuristics or automatic rules [19,36]. Use of heuristics allows laypeople to interpret symptoms more quickly and with less cognitive effort. Heuristics although helpful, often lead to erroneous conclusions [37]. Because the symptoms of the illness vignettes were relatively common, it is possible that inaccurate participants were misled by memories of previous experiences with illness that showed similar, yet distinct patterns of symptoms. Misdiagnosing common symptoms through use of heuristics is similar to the “pattern rule” of the common-sense model where diffuse symptoms are more susceptible to interpretation errors [36]. In contrast, participants who were accurate in their diagnosis may have been relying on more effortful comparison of vignette symptoms and online symptom information. This more effortful matching, even if initially guided by past illness experience, may have been better informed and less reliant on heuristics as these participants were deploying more cognitive resources. Thus, online self-diagnosis may disrupt the reliance on heuristics for symptom interpretation as typically described by the common-sense model. Future work may want to examine what factors predict more effortful processing of symptoms and whether online self-diagnosis can encourage such processing.

Participants who were inaccurate in their online symptom diagnosis also had more difficulty with both the task and the Internet tools. In addition, these participants reported using their home computer for fewer hours per week than accurate participants. Thus, it may be that basic computer skills are predictive of the ability to obtain an accurate online diagnosis. However, inaccurate participants reported being more familiar with the Internet tool to which they were assigned, which would typically suggest experience and more skill. In addition, Sharit and colleagues [24] found that Internet knowledge was related to performance on an information-seeking task although not sufficient to explain performance. Future work may want to obtain measures of performance (eg, speed of diagnosis, cognitive abilities) and computer skills to clarify predictors of accuracy of online symptom diagnosis.

Of note, few participants focused on the source or credibility of the information that they were reviewing, consistent with previous research findings on young and middle-aged adults [13,38]. This may be due to a focus on other activities like Web navigation, which was frequently commented on by participants. Other researchers [39] have used eye-tracking technology to investigate where older adults focus attention during an online health search. This may help to elucidate why credibility was ignored or not verbalized in our sample.

### Implications for Web Design

These findings point to the need for changes and/or updates to current popular health websites. For example, because older adult participants appeared to focus on navigating websites and Web applications, developers may want to update webpages or Web tools with clear navigation aids that guide users as to how the page is structured, how the user can backtrack (ie, return to an earlier viewed page), and how the search bar can best be used. Furthermore, webpages with visual summaries of information, such as tables or figures, may help to decrease effort, allowing users to focus their energies on other aspects of information search.

As few older adult participants noted the credibility of the information source, this seems an important target area. Though past attempts have been made to create user tools for ascertaining the quality and credibility of online information (eg, check lists, website “branding”) [40,41], more effort needs to be made to advise lay searchers of these tools and encourage their use. This would help to ensure that users are gaining access to accurate information via credible sources.

### Limitations

A think-aloud investigation of older adult online health information-seeking produced themes that related to layperson diagnostic strategies, symptom information processing, and especially Web navigation. However, there are factors that limit the generalization of the findings. First, the sample of older adults was predominantly Caucasian, highly educated, and of a comfortable income. In addition, all participants had access to a computer and the Internet at home, suggesting that they already possessed basic computer and Internet skills. While this does limit the generalizability of our findings, previous surveys [6,42] confirm that the majority of adults who search for health information online tend to be of similar backgrounds as our participants (eg, Caucasian, educated, and of higher income). In addition, three-fourths of participants were not familiar with the Internet tool to which they were assigned, and thus, were experiencing the tool for the first time. Nevertheless, a study of older adults in different socioeconomic and geographic locations may demonstrate more variability in the strategies used to find online health information to diagnose symptoms. Furthermore, the study was performed on a university campus that is home to a comprehensive medical center. Thus, our sample of older adults has consistent access to medical care and so may not typically need to search for diagnosis. This may not be true for rural older adults who might lack an easily accessed source of care. Additional investigation as to the type of older adult who engages in online diagnosis may prove beneficial.

### Conclusions

This exploratory study investigated the process of older adult online symptom diagnosis. Few studies have systematically examined this recent phenomenon, especially among older adults. Our findings suggest that, in our sample, older adults tend to rely on hypothetico-deductive matching to diagnose physical symptoms but still may utilize existing medical knowledge and illness experiences to guide diagnosis. This may be because navigating websites and Web tools is a cognitively complex task, providing older adults few resources to sort through the extensive amount of health information online. Thus, additional Web development is necessary to make online search more efficient and accurate for older adult users. In addition, we found that few older adult participants mentioned the credibility of the information that they were viewing. Increased dissemination of previously produced Web tools would be beneficial to ensuring that older adults can access the most appropriate information.

Given the popularity of online self-diagnosis, this study represents the first of its kind in attempting to describe the process that an older patient takes for symptom interpretation. In addition, we focused on a population that is less frequently represented in human-computer interaction studies. While our study provides an initial picture of how some older adults might attempt online self-diagnosis, future work will want to utilize additional study design such as eye-tracking in order to further understand the complex coordination between Web navigation, online symptom information processing, and patient diagnostic strategies.

## Multimedia Appendix 1

Illness vignettes.
