# Supplementary material for: Older Adult Experience of Online Diagnosis: Results From a Scenario-Based Think-Aloud Protocol
Source: J Med Internet Res. 2014 Jan 16;16(1):e16. doi: 10.2196/jmir.2924 (PMC3906693; doi:10.2196/jmir.2924)
Supplement: Supplementary file 1 [file jmir_v16i1e16_app1.pdf]

[Mononucleosis]

**Please read the following story:**

I've been feeling sick for almost a week.  I feel exhausted, and I have a mild fever.  My throat is really sore.  In the past few days, the lymph nodes in my armpits and neck have swollen.  My left side, right below my ribs, is a little sore too.  I wish I would feel better soon.

_____________________________________________________________________________

[Scarlet Fever]

**Please read the following story:**

I've been feeling sick for almost a week. I have a high fever and the lymph nodes in my neck are swollen.  I also have this weird, red rash on my neck and arms.  My tongue has red bumps on it too.  I wish I would feel better soon
